# OCT4 Expression and Vasculogenic Mimicry Formation Positively Correlate with Poor Prognosis in Human Breast Cancer

**DOI:** 10.3390/ijms151119634

**Published:** 2014-10-28

**Authors:** Tieju Liu, Baocun Sun, Xiulan Zhao, Yanlei Li, Qiang Gu, Xueyi Dong, Fang Liu

**Affiliations:** 1 Department of Pathology, Tianjin Medical University, Tianjin 300070, China; E-Mails: liutieju@tmu.edu.cn (T.L.); zhaoxiulan2013@126.com (X.Z.); liyanlei2014@126.com (Y.L.); guqiang2013@126.com (Q.G.); dongxueyi2013a@163.com (X.D.); liufang20145@163.com (F.L.); 2 Department of Pathology, Tianjin Cancer Hospital, Tianjin Medical University, Tianjin 300060, China; 3 Department of Pathology, General Hospital of Tianjin Medical University, Tianjin 300052, China

**Keywords:** OCT4, vasculogenic mimicry, breast cancer

## Abstract

To evaluate the prognostic value of OCT4 expression and vasculogenic mimicry (VM) in human breast cancer, we examined OCT4 expression and VM formation using immunohistochemistry and CD31/PAS (periodic acid-schiff) double staining on 90 breast cancer specimens. All patients were followed up for five–149 months following surgery. Survival curves were generated using Kaplan-Meier method. Multivariate analysis was performed using Cox regression model to assess the prognostic values. Results showed positive correlation between OCT4 expression and VM formation (*p* < 0.05). Both OCT4 expression and VM were also positively correlated with lymph node metastasis, higher histological grade, and Nottingham prognostic index (*p* < 0.05). Patients with OCT4 expression or VM formation exhibited poorer overall survival (OS) and disease-free survival (DFS) than OCT4-negative or VM-negative patients (*p* < 0.05). OCT4-positive/VM-positive patients also had the worst OS and DFS (*p* < 0.05). In multivariate survival analysis, VM, Nottingham prognostic index (NPI), and Her2 were independent prognostic factors related to OS and OCT4-positive/VM-positive patients, whereas NPI and Her2 were independent predictors of DFS. These results suggest that a combined OCT4 expression/VM could improve the prognostic judgment for breast cancer patients.

## 1. Introduction

Breast cancer is the leading cancer among women worldwide, with a higher increase rate for young patients [1]. Breast cancer is also a heterogeneous disease; its tumors have the same clinical, pathological, and hormone receptor statuses but may have different metastatic potentials because of inherently dissimilar biological characteristics. Although some pathological factors (for example, histological type and grade) and hormone receptors, including ER, PR, and Her2, have been widely used as reference in clinical diagnosis and treatment, their prognostic values for breast cancer still have certain limitations. Therefore, identification of reliable molecular prognostic markers is important in clinical practice for breast cancer treatment.

OCT4 is a nuclear transcription factor of the POU-homeodomain family that has critical roles in self-renewal, pluripotency, and lineage commitment of embryonic stem cells (ESCs) [2]. During embryogenesis, OCT4 is expressed in primordial germ cells, oocytes, and pre-implantation embryos and then restricted to the inner cell mass of the blastocyst [3]. Ablation of OCT4 function in human ESCs leads to differentiation into trophectoderm [4]. A recent study demonstrating that somatic cells can acquire ESC properties via dedifferentiation or reprogramming of combined transcription factors, namely, OCT4, SOX2, KLF4, and c-MYC, has provided a new insight into cancer stem cells (CSCs) [5]. Each of the reprogramming factors has established roles in oncogenesis; OCT4 plays a dynamic role in initiating germ cell tumors and has been proposed as a useful marker for seminomas, embryonal carcinomas, and other germ cell tumors [6]. Although OCT4 is highly expressed in seminomas, other tumors of non-germcellorigin show detectable levels compared with their normal cell counterparts, such as esophageal cell carcinoma [7] and prostate cancer [8]. Kim *et al.* showed that OCT4 alone is sufficient to directly reprogram adult mouse neural stem cells to pluripotent stem cells [9]. OCT4 is also a core regulator of stem cell self-renewal and differentiation, which was recently validated as a CSC target [10]. Clinical studies have shown that OCT4 is a CSC marker, and its intense expression is associated with further disease progression, greater metastasis, and shorter cancer-related survival compared with those of tumors with moderate and low OCT4 expression [11].

Tumors require blood supply for growth and distant dissemination. Much attention has been focused on the role of angiogenesis—the recruitment of new vessels into a tumor from pre-existing vessels. However, angiogenesis may not be the only mechanism by which tumors acquire a microcirculation. In 1999, vasculogenic mimicry (VM) was demonstrated as a phenomenon in which highly aggressive and metastatic melanoma cells can form highly patterned vascular channels *in vitro* composed of a basement membrane stained positive with periodic acid-schiff (PAS) in the absence of endothelial cells and fibroblasts [12]. Since the introduction of VM in 1999 as a novel paradigm for tumor perfusion, many studies have contributed new insights into the underlying molecular pathways supporting VM [13]. VM could be demonstrated as an example of the remarkable plasticity displayed by aggressive melanoma cells, which suggests that these tumor cells have acquired an embryonic-like phenotype.

CSCs are cancer cells (found within tumors or hematological cancers) that possess characteristics associated with normal stem cells, specifically the ability to induce all cell types found in a particular cancer sample. Human malignancies harbor a subpopulation of CSCs that fuel tumor growth, local invasion, and distant metastasis formation. Bian *et al.* [14] proposed a concept of CSC plasticity, in which CSCs possess powerful capacity in self-renewal and multipotent differentiation. These CSC subsets form branching lumens and tubes to provide nutrition for tumor mass by differentiation/transdifferentiation, thereby resembling VM networks [14]. Our previous study also showed that CSCs can participate in VM formation, CSC subpopulation inside triple-negative breast cancer can organize VM, and transdifferentiative capacity of CSCs might be needed [15].

Although OCT4 is expressed in CSCs and CSCs are involved in VM formation based on our previous study [15], the correlation of OCT4 expression and VM in human breast cancer and the relevance of their co-existence within clinical parameters remain unclear. In the current study, expression patterns of OCT4 and VM were examined by immunohistochemistry (IHC) on 90 samples of human breast cancer cases. The correlation of OCT4 expression and VM and its relevance to clinicopathologic parameters were explored. Prognostic roles of OCT4 expression and VM in human breast cancer were also evaluated using Cox regression and Kaplan-Meier analysis. To our knowledge, this study is the first to report the correlation of OCT4 expression and VM and its clinical significance for breast cancer patients.

## 2. Results

### 2.1. OCT4 Expression and VM Formation in Breast Cancer Specimens

**Figure 1:**
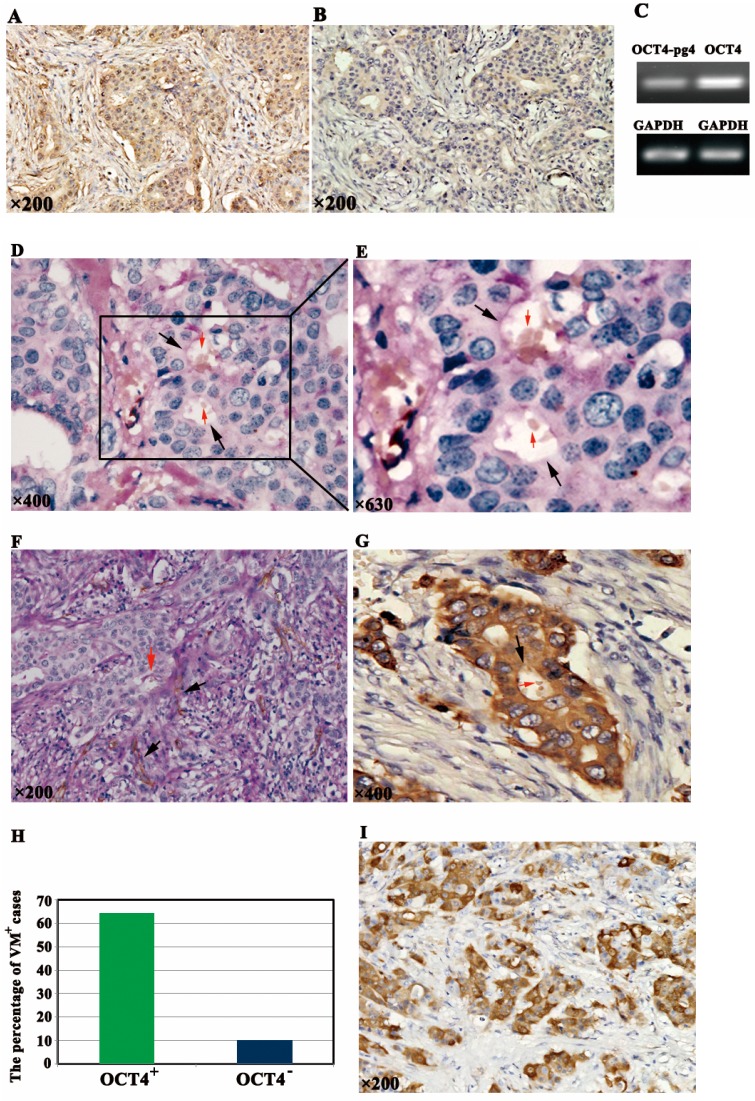
OCT4 expression and vasculogenic mimicry (VM) formation in breast cancer specimens. (**A**) Positive expression of OCT4 was mainly localized in the nuclei of cancer cells; (**B**) Negative expression of OCT4 in breast cancer; (**C**) RT-PCR showed that the relative mRNA expression level of *OCT4* was dramatically higher than that of *OCT4-pg4*. Glyceraldehyde3-phosphate dehydrogenase (*GAPDH*) was used as an endogenous control; (**D**) VM formation in breast cancer tissue (black arrows indicate VM channels formed by tumor cells; red arrows indicate red blood cells); (**E**) VM is shown in higher magnification (black arrows indicate VM channels formed by tumor cells; red arrows indicate red blood cells); (**F**) CD31^+^blood vessels and VM (black arrows indicate typical blood vessels with brown CD31^+^ staining; red arrows indicate one VM structure with CD31-negative expression); (**G**) Cells lining VM were positive for CK staining (black arrow indicate VM and red arrow indicate red blood cells); (**H**) OCT4-positive expression directly correlated with VM formation in breast cancer samples; and (**I**) Positive expression of ALDH1 was located in the cytoplasm of cancer cells.

To investigate OCT4 expression in breast cancer, IHC staining was performed on 90 breast cancer tissue sections and the OCT4 antibody recognized isoform A and B of *OCT4* gene. Positive signals of OCT4 were mainly localized in the nuclei of cancer cells (Figure 1A). By contrast, negative OCT4 expression is shown in Figure 1B. At least 10 fields in each specimen were randomly selected and examined under high-power magnification, and >500 cells were counted to determine the percentage of positive cells. Based on the criteria established by Fan *et al.* [16] with minor modifications, ≥10% percentage of positive cells were considered to have OCT4-positive expression.

According to the literature, the human *OCT4* has six pseudogenes (pg), of which *OCT4-pg4* was more often found to be transcribed in somatic cancers [17]. In order to exclude the *OCT4-pg4* expression and verify that the OCT4 protein expression is due to *OCT4* gene expression, reverse-transcription polymerase chain reaction (RT-PCR) was performed to examine the expression levels of *OCT4* gene and *OCT4-pg4* in breast cancer tissue with positive OCT4 protein expression detected by IHC. The relative mRNA expression level of *OCT4* was dramatically higher than that of *OCT4-pg4* (Figure 1C). Thus, the result demonstrated that in our study the positive OCT4 protein expression was mainly the consequence of *OCT4* gene overexpression.

Our previous study [15] demonstrated that CD31 and PAS histochemical and immunohistochemical double staining could recognize VM in tumor tissues. CD31-negative, PAS-positive, and vascular-like patterns containing red blood cells, which were formed by breast cancer cells, were identified as VM. Breast cancer cells mimic endothelial cells to form extracellular matrix-rich channels (PAS-positive) without necrosis and inflammatory cells infiltrating around the channels (Figure 1D,E; black arrows indicate VM, red arrows indicate red blood cells). Typical blood vessels showed positive reaction for CD31 on their luminal surface and PAS-positive reaction on their wall (Figure 1F; black arrows indicate CD31^+^ blood vessels, red arrows indicate VM). Cells lining VM were positive for cytokeratin (CK) staining (Figure 1G; black arrow indicate VM and red arrow indicate red blood cells).

Of the 90 cases analyzed, 31 (34.4%) were positive for OCT4 and 26 (28.6%) for VM formation. Among them, 20 were positive for both markers, 53 were both negative, 11 were OCT4 positive only, and six were VM positive only. Interestingly, statistical analysis revealed that OCT4-positive expression is directly correlated with VM formation in these samples (*R* = 0.570, *p* = 0.000) (Figure 1H).

Breast CSC marker aldehyde dehydrogenase 1 (ALDH1) expression was also detected, and the positive signals were located in the cytoplasm (Figure 1I). About ≥10% percentage of positive cells were considered ALDH1-positive expression. Of the 90 cases analyzed, 40 (44.4%) were positive for ALDH1 expression. Twenty-three were positive for both ALDH1 and OCT4 expression, 42 were both negative, eight were OCT4 positive only, and 17 were ALDH1 positive only. Notably, statistical analysis revealed that OCT4-positive expression is directly correlated with ALDH1-positive expression (*R* = 0.434, *p* = 0.000).

### 2.2. Association of OCT4-Positive Expression and VM with Clinicopathological Characteristics

Nottingham prognostic index (NPI) is used to determine prognosis following surgery for breast cancer. NPI value is calculated using three pathological criteria: size of lesion, number of lymph nodes involved, and grade of tumor. The index is calculated using the formula NPI = (0.2 × *S*) + *N* + *G* (*S* is the size of the lesion in centimeters; *N* is the number of lymph nodes involved: 0 = 1, 1–3 = 2, >3 = 3; *G* is the grade of tumor: Grade I = 1, Grade II = 2, Grade III = 3). In our study, NPI ranged from 2.20–7.00 (mean 4.43). In accordance with the study of Fernando Schmitt [18], NPI can stratify breast cancer patients into good (NPI < 3.4), moderate (3.4 ≤ NPI ≤ 5.4), and worse prognosis (NPI > 5.4). Both OCT4 and VM were not only correlated with lymph node metastasis (*p* = 0.022 and *p* = 0.004) and histological grade (*p* = 0.038 and *p* = 0.000), but also with NPI (*p* = 0.006 and *p* = 0.000) (Table 1). However, no significant association existed in OCT4 or VM with other clinicopathological features.

**Table 1:** Relationship between clinicopathological variables and OCT4 expression or VM formation.

Variables	OCT4 Expression				VM Formation		
	Cases (*n*)	+	−	*p*	+	−	*p*
**Age (years)**				0.212			0.508
<50	47	19	28		15	32	
≥50	43	12	31		11	32	
**Tumor size**				0.864			0.656
T1: <2	30	10	20		9	21	
T2: 2–5 cm	56	19	37		15	41	
T3: >5 cm	4	2	2		2	2	
**Nodal status**				0.022 *			0.004 *
0	49	11	38		8	41	
1–3	17	7	10		5	12	
≥4	24	13	11		13	11	
**Grade**				0.038 *			0.000 *
I	23	4	19		0	23	
II	30	9	21		6	24	
III	37	18	19		20	17	
**NPI**				0.006 *			0.000 *
NPI < 3.4	26	4	22		1	25	
3.4 ≤ NPI ≤ 5.4	42	14	28		9	33	
NPI > 5.4	22	13	9		16	6	
**ER**				0.391			0.630
Positive	52	16	36		14	38	
Negative	38	15	23		12	26	
**PR**				0.321			0.499
Positive	50	15	35		13	37	
Negative	40	16	24		13	27	
**Her2**				0.114			0.247
0/+	46	18	28		14	32	
++	20	3	17		3	17	
+++	24	10	14		9	15	

* Statistically significant.

### 2.3. Correlation of OCT4-Positive Expression and VM with Overall Survival (OS) and Disease-Free Survival (DFS)

**Figure 2:**
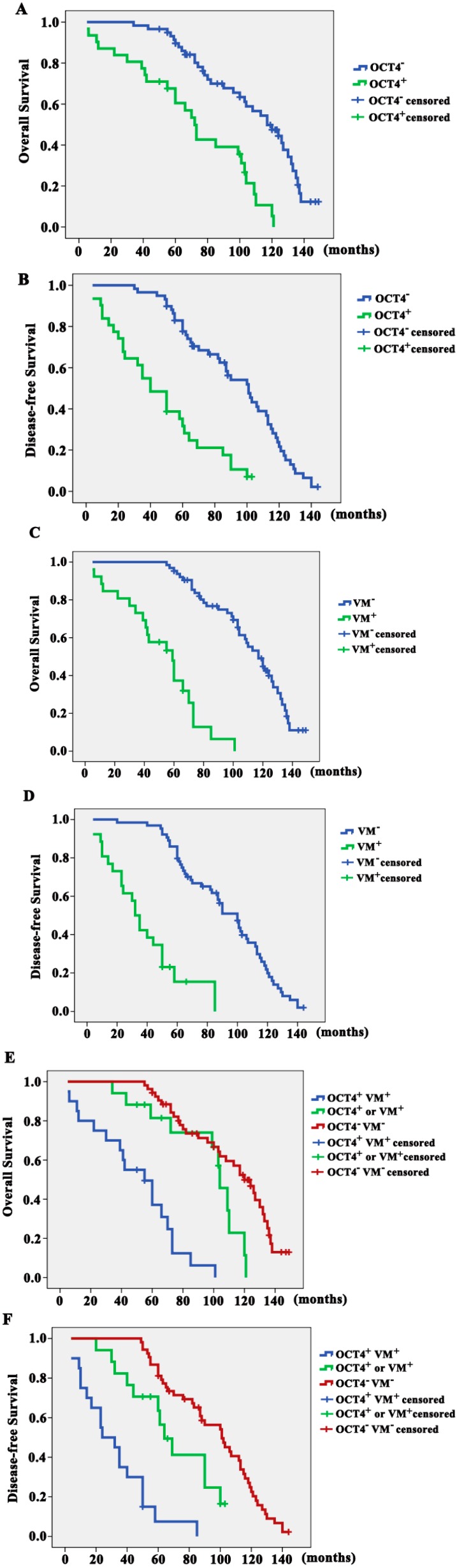
Correlation of OCT4-positive expression and VM with overall survival (OS) and disease-free survival (DFS). (**A**,**B**) OCT4-positive patients showed poorer prognosis for OS and DFS than OCT4-negative patients; (**C**,**D**) Patients with VM displayed poorer prognosis for OS and DFS than those without VM; and (**E**,**F**) Patients with both OCT4-positive expression and VM formation exhibited the worst survival. By contrast, patients with both negative OCT4 and VM demonstrated the highest survival for OS and DFS.

In this study, survival analysis revealed that OCT4-positive patients showed poorer prognosis for OS and DFS than those with OCT4-negative expression (Figure 2A,B). The mean (95% CI) OS time and mean DFS time were 71.141 (57.787–84.495) months and 46.880 (35.904–57.855) months, respectively, for OCT4-positive patients, whereas the corresponding mean OS time and mean DFS time were 108.534 (99.728–117.341) months and 93.430 (85.279–101.582) months for OCT4-negative patients (*p* = 0.000). Consistently, patients with VM displayed poorer prognosis for OS and DFS (Figure 2C,D) than patients without VM. The mean OS time and mean DFS time were 52.140 (41.386–62.893) months and 38.154 (27.913–48.395) months, respectively, for patients with VM, whereas the corresponding mean OS time and mean DFS time were 111.323 (104.149–118.498) months and 92.482 (84.995–99.968) months for patients without VM (*p* = 0.000). Notably, patients with both OCT4-positive expression and VM formation exhibited the worst survival. By contrast, patients with both negative OCT4 and VM demonstrated the highest survival for OS and DFS (Figure 2E,F).

To analyze the relevance of OCT4-positive expression, VM formation, and clinicopathological features with OS and DFS, univariate Cox regression analysis was performed with factors including lymph node status, histological grade, NPI, OCT4-positive expression, VM formation. Combined OCT4-positive expression and VM formation was also analyzed (Table 2 and Table 3). Statistical analysis indicated that either OCT4-positive expression or VM formation was significantly associated with poor OS and DFS (*p* = 0.000). More importantly, the combined OCT4-positive expression and VM formation was significantly associated with poor OS and DFS (*p* = 0.000). In multivariate analysis, VM formation (*p* = 0.000; HR = 7.744; CI 3.514–17.069), NPI (*p* = 0.008; HR = 1.903; CI 1.187–3.050), and Her2 (*p* = 0.031; HR = 1.398; CI 1.031–1.894) were independent prognostic factors related to OS (Table 2). The combined OCT4-positive expression and VM formation (*p* = 0.000; HR = 7.880; CI 3.864–16.067), NPI (*p* = 0.001; HR = 1.895; CI 1.288–2.789), and Her2 (*p* = 0.013; HR = 1.424; CI 1.078–1.880) were independent predictors of DFS (Table 3). However, OCT4-positive expression did not show independent prognostic significance for OS and DFS in the multivariate analysis (*p* > 0.05).

**Table 2:** Cox proportional-hazard regression model analysis for OS.

Variables	Univariate Analysis			Multivariate Analysis		
	HR **	95% CI	*p*	HR **	95% CI	*p*
**Age (years)**						
<50, ≥50	0.956	0.575–1.587	0.861			
**Tumor size**						
T1, T2, T3	0.931	0.575–1.507	0.771			
**Nodal status**						
0, 1–3, ≥4	1.625	1.178–2.241	0.003 *			
**Grade**						
I, II, III	2.013	1.427–2.840	0.000 *			
**NPI**						
Good, Moderate, Worse	2.837	1.870–4.303	0.000 *	1.903	1.187–3.050	0.008 *
**ER**						
Positive, Negative	0.640	0.386–1.061	0.083			
**PR**						
Positive, Negative	0.724	0.438–1.197	0.208			
**Her2**						
0/+, ++, +++	1.210	0.897–1.632	0.211	1.398	1.031–1.894	0.031 *
**OCT4**						
Positive, Negative	3.459	1.974–6.061	0.000 *			
**VM**						
Positive, Negative	10.750	5.404–21.383	0.000 *	7.744	3.514–17.069	0.000 *
**OCT4/VM**						
OCT4^+^/VM^+^, Non-OCT4^+^/VM^+^	8.907	4.565–17.378	0.000 *			

* Statistically significant; HR **: Hazard ratio.

**Table 3:** Cox proportional-hazard regression model analysis for DFS.

Variables	Univariate Analysis			Multivariate Analysis		
	HR **	95% CI	*p*	HR **	95% CI	*p*
**Age (years)**						
<50, ≥50	0.997	0.634–1.568	0.989			
**Tumor size**						
T1, T2, T3	1.034	0.671–1.592	0.881			
**Nodal status**						
0, 1–3, ≥4	1.517	1.137–2.025	0.005 *			
**Grade**						
I, II, III	1.786	1.314–2.426	0.000 *			
**NPI**						
Good, Moderate, Worse	2.339	1.642–3.331	0.000 *	1.895	1.288–2.789	0.001 *
**ER**						
Positive, Negative	0.753	0.476–1.192	0.227			
**PR**						
Positive, Negative	0.673	0.426–1.062	0.089			
**Her2**						
0/+, ++, +++	1.228	0.935–1.612	0.139	1.424	1.078–1.880	0.013 *
**OCT4**						
Positive, Negative	4.097	2.407–6.973	0.000 *			
**VM**						
Positive, Negative	8.557	4.509–16.238	0.000 *			
**OCT4/VM**						
OCT4^+^/VM^+^, Non-OCT4^+^/VM^+^	10.011	5.228–19.170	0.000 *	7.880	3.864–16.067	0.000 *

* Statistically significant; HR **: Hazard ratio.

## 3. Discussion

Breast cancer is the most common disease in women that seriously endangers their health. In routine clinical practice, common information including histological grade, tumor stage and size, lymph node metastasis status, ER, PR, and Her2 expression status are obtained for each patient. However, the sometimes clinician cannot obtain enough prognostic and therapeutic data from traditional information. This phenomenon indicates that breast cancer is a heterogeneous group of tumors that has diverse biologic behavior, outcomes, and treatment responses [19].

VM, a newly defined pattern of tumor blood supply, signifies the functional plasticity of aggressive cancer cells forming vascular networks [15,20]. As an angiogenesis-independent mechanism, VM could provide a special passage without endothelial cells for a tumor blood supply that can directly or indirectly interact with other vasculatures [13,21]. VM could serve as a selective advantage for rapidly growing tumors in need of perfusion. Tumors exhibiting VM are usually related to more aggressive tumor biology and increased tumor-related mortality. VM in prostate cancer is an independent marker of poor prognosis [22]. Considerable research has shown that the presence of VM in a highly invasive tumor is associated with high tumor grade, invasion and metastasis, and short survival in malignant tumor specimens [15,20,21,23]. *OCT4* constitutes part of an important gene regulatory network and is essential for embryogenesis and/or pluripotency and self-renewal of ESCs [24]. Ezeh *et al.* [25] also found that normal breast tissues do not express detectable levels of OCT4, and breast carcinoma in advanced stage reveals OCT4 expression along with other stem cell markers. Chang *et al.* [26] demonstrated that OCT4 promotes tumorigenesis of colorectal cancer cells in both autocrine and paracrine manners. Saigusa *et al.* [27] showed that OCT4 expression is associated with the recurrence of rectal cancer after chemoradiotherapy.

In the current study, we examined OCT4 expression and VM formation in 90 human breast cancer specimens using IHC. To our knowledge, this study is the first to present clinical evidence indicating that OCT4 expression and VM formation are positively correlated in human breast cancer. OCT4 is recognized as an ESC-specific protein and has been frequently described as a CSC marker in cervical cancer [28], oral squamous cell carcinomas [29], and breast cancer [30]. Our previous study [15] showed that breast CSCs may participate in VM formation. In the current study, the correlation of OCT4-positive expression and VM formation suggested that OCT4 could exert a promoting role in VM formation by boosting CSC subpopulation, thereby potentiating breast cancer metastasis.

We found that patients with VM or OCT4-positive expression showed poorer OS and DFS than VM-negative or OCT4-negative patients. Kaplan-Meier analyses also revealed that OCT4-positive expression combined with VM formation significantly correlated with the worst OS and DFS in breast cancer. Patients in the VM-positive/OCT4-positive group showed the worst survival, whereas those in the VM-negative/OCT4-negative group exhibited the highest survival. These results indicated that co-existence of VM and OCT4 expression predicted worst survival and may serve as the key molecular prognostic indicator for breast cancer survival.

In this study, the relationship of the clinicopathological factors with VM and OCT4 expression was analyzed. This retrospective study of 90 breast cancer patients showed that both OCT4-positive expression and VM formation had strong correlation with lymph node metastasis, higher histological grade, and NPI. Node metastasis is one of the typical indicators of breast cancer metastasis. A previous study reported that high expression of OCT4 is correlated with liver metastasis in human colorectal cancer [31]. Recent research reported that VM contributes to lymph node metastasis of laryngeal squamous cell carcinoma [32], and patients with breast carcinomas displaying VM have a higher rate of distant metastasis (liver, lungs, and bone) [33]. Collectively, these findings suggest that the increased OCT4 expression in breast cancer cells promotes CSC subpopulation and VM formation, thereby facilitating tumor cell migration and metastasis into the blood and lymphatic vessels and promoting breast cancer aggressiveness.

We also found that the proportion of both OCT4-positive expression and VM formation increased with the increase in histological grade, which was consistent with a previous study on hepatocellular carcinoma and glioma [34,35]. VM is an alternative type of blood supplement formed by highly invasive and genetically dysregulated tumor cells with a pluripotent embryonic-like genotype. Therefore, we conclude that breast cancer cells with higher histological grade have more cell heteromorphism and can display cancer plasticity by genetic reversion to a pluripotent embryonic-like genotype to ultimately form VM. Consequently, NPI value is higher in OCT4-positive or VM-positive cells than in OCT4-negative or VM-negative cells, which also supports the conclusion that breast cancer with OCT4-positive expression and VM formation indicates poor prognosis.

Consistent with previous reports, our Cox multivariate analysis demonstrated that VM was an independent prognostic factor for OS, and the co-existence of VM formation and OCT4-positive expression was an independent prognostic factor for DFS in breast cancer patients. These findings strongly suggest that combined VM formation and OCT4-positive expression may serve as a poor prognosis indicator for breast cancer survival. The relationship of OCT4-positive expression and VM formation might suggest a feasible therapeutic strategy for targeting OCT4 expression in breast cancer with VM formation.

## 4. Materials and Methods

### 4.1. Tissue Specimens

Tissue collection and analysis in this study were approved by the Ethical Committee of Tianjin Medical University, China. All subjects gave their informed consent for inclusion before they participated in the study. The study was conducted in accordance with the Declaration of Helsinki, and the protocol was approved by the Ethics Committee of Tianjin Medical University. All breast surgical specimens were formalin fixed and paraffin embedded, and then anonymized after collection from the archival file of the Pathology Department of Tianjin Medical University. Ninety cases of invasive ductal carcinoma were randomly selected from 1998 to 2005. The pathologic diagnosis was reviewed by two senior pathologists based on hematoxylin and eosin-stained sections according to the 2003 WHO histological classification of breast tumors. All the clinicopathological parameters, including age, histological grade, lymph node metastasis status, tumor size, ER, PR, and Her2 status, were obtained from the documents. Histological grade was assessed using Bloom-Richardson scale in Elston-Ellis modification.

All of the patients were followed up by clinic interview or phone call. The total follow-up period was 5–149 months (median was 86.611 months). OS time was calculated as the duration from the date of surgery to the date of death. DFS time was calculated as the duration from the date of surgery to the date of documented disease progression (breast cancer-derived relapse/metastasis).

### 4.2. Immunohistochemical and Histochemical Double-Staining Methods

Tissue sections (4–5 μm) were deparaffinized and hydrated following standard procedures. Immunostaining was performed using the super-sensitivity S-P IHC kit (Beking Zhongshan Golden Bridge Biotechnology Limited Company, Beijing, China). After immersing in 3% H_2_O_2_ for 10 min to eliminate endogenous peroxidase, the sections were microwaved for antigen retrieval in 0.01 M sodium citrate for 15 min. After blocking in 10% goat serum for 30 min and incubation with primary antibodies against proteins, namely, OCT4 (1:100; Santa Cruz Biotechnology, Santa Cruz, CA, USA) and CD31 (Beking Zhongshan Golden Bridge Biotechnology Limited Company, Beijing, China) at 4 °C overnight, the tissue sections were incubated with appropriate biotin-labeled secondary antibodies, followed by peroxidase-conjugated avidin (Beking Zhongshan Golden Bridge Biotechnology Limited Company, Beijing, China). Positive signals were developed in the 3,3-diaminobenzidine tetrahydrochloride solution. After counterstaining with hematoxylin or PAS, the slides were ready for microscopic examination.

Samples of breast carcinoma with high expression of OCT4 or VM formation served as the positive control. Negative control sections were treated with species-matched, normal non-immune IgG instead of the primary antibody.

### 4.3. Reverse-Transcription Polymerase Chain Reaction (RT-PCR) Analysis

Total RNA from frozen breast cancer tissue with positive OCT4 protein expression was extracted by using Trizol reagent (Invitrogen, Waltham, MA, USA) according to the manufacturer’s instructions. Based on the study Wang *et al.* [17] performed, the primer sequences were listed as follows: *OCT4*: forward 5'-GATGGCGTACTGTGGGCCC-3' and reverse 5'-TGGGACTCCTCCGGGTTTTG-3'; *OCT4-pg4*: forward 5'-CAGAAACCCTCTTGCAGGCT-3' and reverse 5'-GAACCACACTCGGACCACAT-3'; *GAPDH* forward 5'-CCTGGCCAAGGTCATCCATGAC-3', and reverse 5'-TGTCATACCAGGAAATGAGCTTG-3'. Finally PCR products were run on a 1.5% (*w*/*v*) agarose gel.

### 4.4. Statistical Analysis

The correlation between OCT4 expression and VM formation was analyzed using Spearman’s rank test. The relationship between OCT4 expression or VM formation and the clinicopathologic parameters was analyzed using two-tailed Chi-square test or Monte Carlo method. Survival curves were estimated using Kaplan-Meier method and compared by log rank test. Univariate or multivariate analysis of prognostic factors was tested for Cox proportional-hazard regression models. All statistical analyses were performed using the SPSS software system (version 16.0; SPSS, Chicago, IL, USA). *p* < 0.05 was considered statistically significant.

## 5. Conclusions

Our research is the first to demonstrate that VM formation and OCT4-positive expression are directly correlated in human breast cancer. We also confirm that VM formation and OCT4-positive expression are prognostic factors in human breast cancer. Moreover, the combined co-existence of OCT4-positive/VM could improve the prognostic judgment for breast cancer patients.
